# Supplementary material for: Synergistic Neuroprotective Effect of Schisandra chinensis and Ribes fasciculatum on Neuronal Cell Death and Scopolamine-Induced Cognitive Impairment in Rats
Source: Int J Mol Sci. 2019 Sep 12;20(18):4517. doi: 10.3390/ijms20184517 (PMC6770047; doi:10.3390/ijms20184517)
Supplement: Supplementary file 1 [file ijms-20-04517-s001.zip › Supplementary files/Supplementary Materials with track change.docx]

**Supplementary Figures**

**Synergistic neuroprotective effect of *Schisandra chinensis* and *Ribes fasciculatum* on neuronal cell death and scopolamine-induced cognitive impairment in rats**

Eunkuk Park, Min Jeong Ryu, Nam Ki Kim, Mun Hyoung Bae, Youngha Seo, Jeonghyun Kim, , Subin Yeo, Memoona Kanwal, Chun Whan Choi, Jun Young Heo and Seon-Yong Jeong


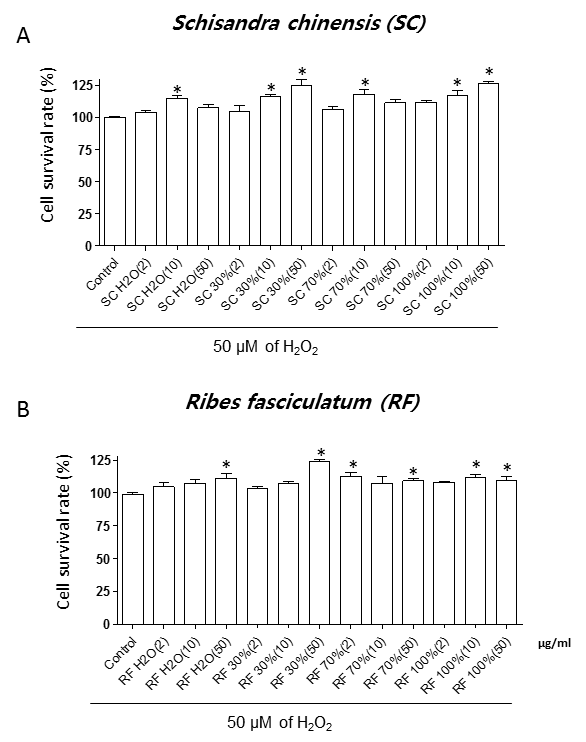


**Figure S1.** The effect of different ethanol extracts of SC and RF on cell survival rate. SC and RF were extracted with four different concentrations of ethanol (0%, 30%, 70%, or 100%) and PC12 neuronal cells were treated with 2, 10, or 50 μg/ml of SC or RF extracts. The protective effect of the extracts was measured by the WST assay. Control is a treatment of 50 µM of H_2_O_2_. The data shown are means ± SEM. *: *p* < 0.05 vs. control.


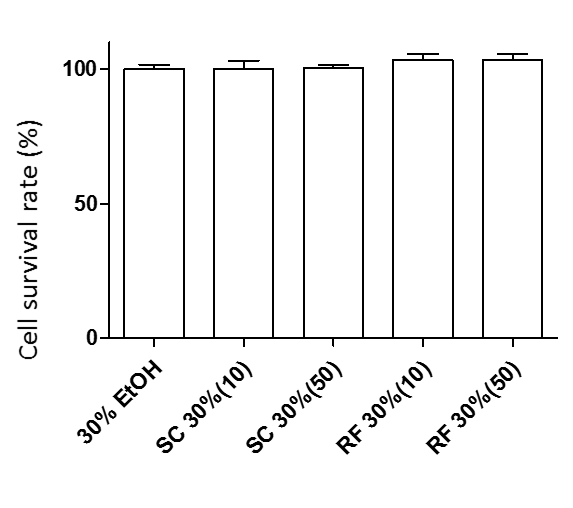


**Figure S2.** SC and RF did not affect the proliferation of neuronal cells. The cells were cultured with SC or RF (10 or 50 μg/ml), and cell viability was analyzed.


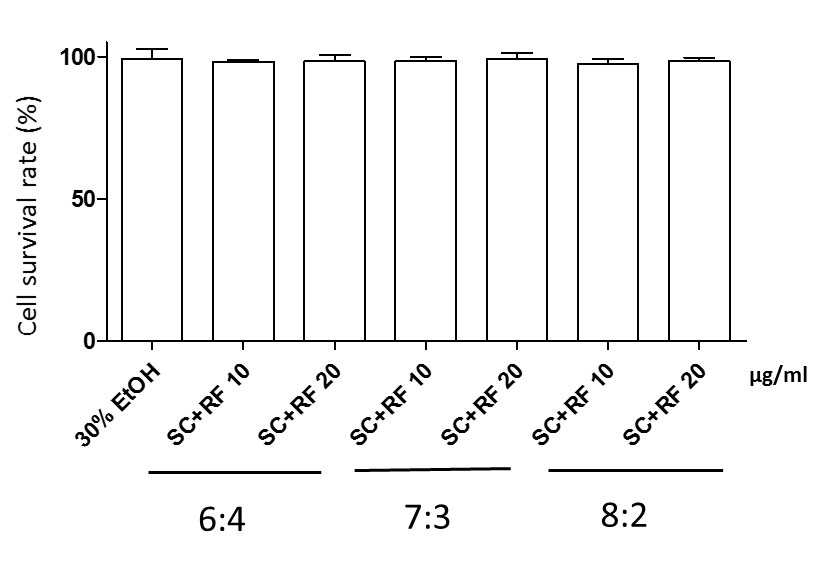


**Figure S3.** A combination of SC and RF did not affect PC12 cell proliferation. The cells were treated with different ratios (6:4, 7:3, or 8:2) of a combination of SC and RF (10 or 50 μg/ml), and cell viability was analyzed.
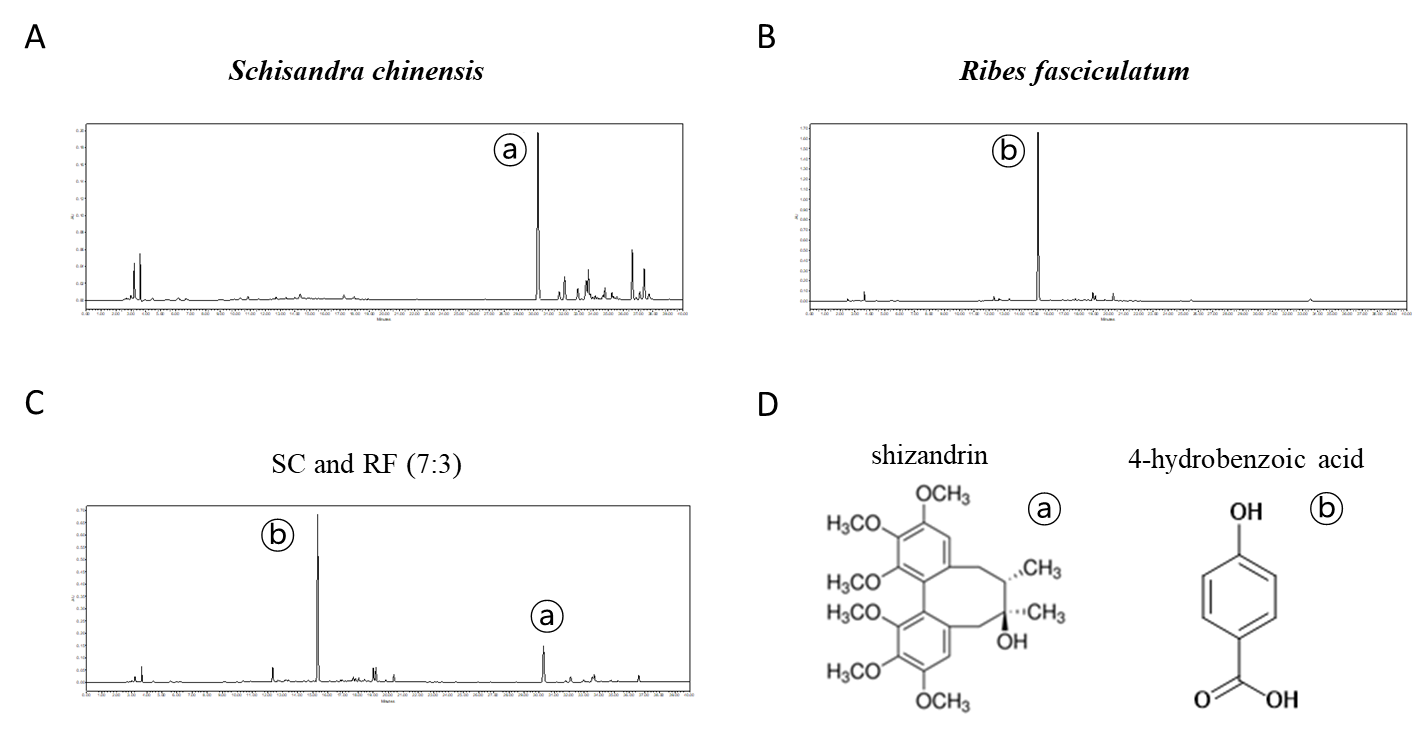


**Figure S4.** HPLC profiles of the SC (A) and RF (B) and combination of SC and RF (7:3) (C) extracts obtained by HPLC-ESI-MS analysis Chemical structures (D) of the main constituent of shizandrin ⓐ identified in SC and 4-hydrobenzoic acid ⓑ identified in RF.
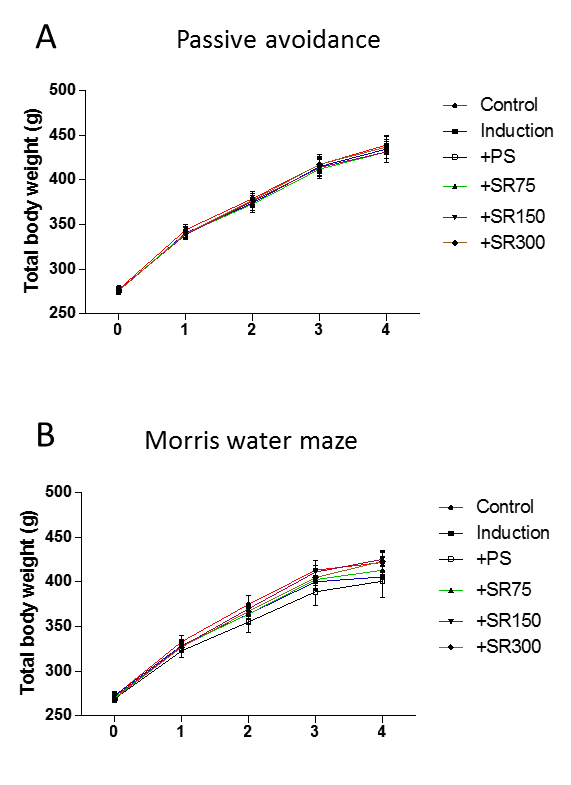


**Figure S5.** Total body weight changes during the passive avoidance test (A) and the Morris water maze (B) experiments. Rats were treated orally with SR (SC+RF) (75, 150 or 300 mg/kg/day) for 23 days and memory impairment was measured by the Morris water maze and the passive avoidance test for five days. Total body weight was measured in each week. Abbreviation: PS, phosphatidylserine.


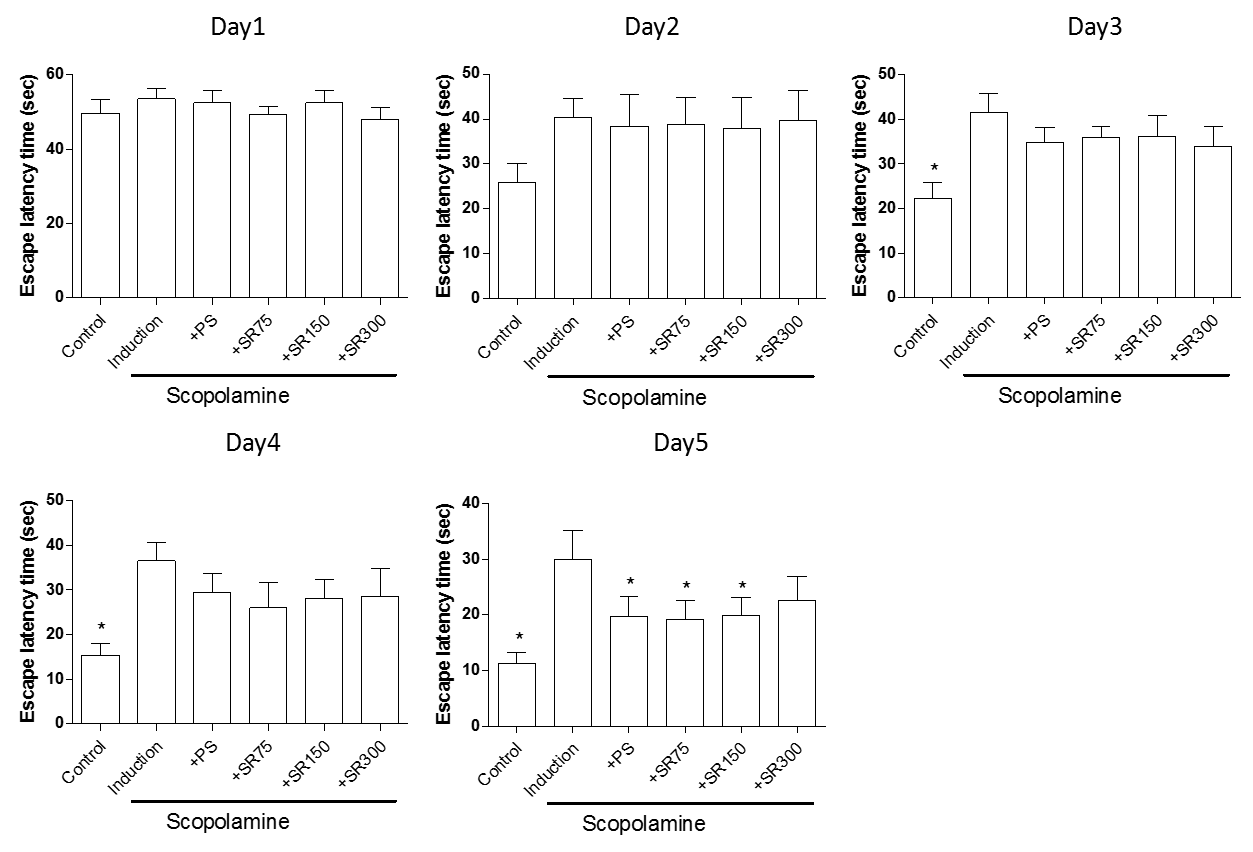


**Figure S6.** Changes in escape latency time over 5 days in scopolamine-treated rats. Seven-week-old male Sprague Dawley® rats were administered phosphatidylserine orally and a mixture of SR (SC+RF) for 23 days before scopolamine injection. Escape latency time was measured daily for 5 days. Abbreviation: PS, phosphatidylserine.
