# Supplementary material for: Synergistic Neuroprotective Effect of Schisandra chinensis and Ribes fasciculatum on Neuronal Cell Death and Scopolamine-Induced Cognitive Impairment in Rats
Source: Int J Mol Sci. 2019 Sep 12;20(18):4517. doi: 10.3390/ijms20184517 (PMC6770047; doi:10.3390/ijms20184517)
Supplement: Supplementary file 1 [file ijms-20-04517-s001.zip › Supplementary files/Supplementary Table.docx]

**Supplementary Table 1**. Results of water-soluble tetrazolium salt (WST) assay before/after treatment of H_2_O_2_ in the ethanol extracts of the 42 plants native to Korea in PC12 cells. Extracted all Korean plants with 30% EtOH.

| **Extracts** | | **Conc.** | **Survival rate before H_2_O_2_** | **H_2_O_2_-teated cells survival rate** | **Extracts** | | **Conc.** | **Survival rate before H_2_O_2_** | **H_2_O_2_-teated cells survival rate** | **Extracts** | | **Conc.** | **Survival rate before H_2_O_2_** | **H_2_O_2_-teated cells survival rate** |
| --- | --- | --- | --- | --- | --- | --- | --- | --- | --- | --- | --- | --- | --- | --- |
| **No.** | **Name** | **㎍/ml** |  |  | **No.** | **Name** | **㎍/ml** |  |  | **No.** | **Name** | **㎍/ml** |  |  |
| **1** | ***Rehmanniae Radix Preparata*** | **10** | **100.1** | **99.8** | **2** | ***Lithospermi Radix*** | **10** | **101.3** | **112.8** | **3** | ***Salicornia Herbacea*** | **10** | **100.6** | **94.4** |
|  |  | **20** | **100.2** | **98.4** |  |  | **20** | **101.3** | **108.6** |  |  | **20** | **100.53** | **100.8** |
| **4** | ***Rhizoma Drynariae*** | **10** | **101.3** | **116.2** | **5** | ***Lycii Radicis Cortex*** | **10** | **102.2** | **99.1** | **6** | ***Radix Polygoni Multiflori*** | **10** | **101.5** | **108.3** |
|  |  | **20** | **101.4** | **99.47** |  |  | **20** | **101.7** | **104** |  |  | **20** | **93.23** | **120.4** |
| **7** | ***Ribes fasciculatum*** | **10** | **102.2** | **132.2** | **8** | ***Nelumbinis Semen*** | **10** | **101.5** | **106** | **9** | ***Scutellaria Baicalensis*** | **10** | **97.7** | **100** |
|  |  | **20** | **102.4** | **134.3** |  |  | **20** | **101.6** | **118.6** |  |  | **20** | **97.76** | **116.8** |
| **10** | ***Fructus Psoraleae*** | **10** | **101.7** | **98.84** | **11** | ***Paeoniae Radix*** | **10** | **97.78** | **124.3** | **12** | ***Curcuma Aromatica Salisb.*** | **10** | **98.2** | **113.8** |
|  |  | **20** | **88.68** | **108.7** |  |  | **20** | **97.84** | **102.2** |  |  | **20** | **98.26** | **104** |
| **13** | ***Acori Graminei Rhizoma*** | **10** | **97.86** | **115** | **14** | ***Morus Alba L.*** | **10** | **98.41** | **104.5** | **15** | ***Fructus Aurantii Immaturus*** | **10** | **102.7** | **111.7** |
|  |  | **20** | **98.08** | **113.5** |  |  | **20** | **98.66** | **116.9** |  |  | **20** | **94.38** | **102.7** |
| **16** | ***Eucommia Ulmoides*** | **10** | **98.72** | **95.82** | **17** | ***Herba Cirsii*** | **10** | **94.64** | **100.2** | **18** | ***Caragana Sinica*** | **10** | **98.98** | **95.69** |
|  |  | **20** | **98.84** | **99.26** |  |  | **20** | **94.71** | **99.7** |  |  | **20** | **99.11** | **93.3** |
| **19** | ***Kalopanacis Cortex*** | **10** | **94.84** | **98.41** | **20** | ***Rosae Laevigatae Fructus*** | **10** | **99.13** | **94.8** | **21** | ***Glycine Semen Preparatum*** | **10** | **95.44** | **100.8** |
|  |  | **20** | **94.98** | **94.38** |  |  | **20** | **99.26** | **106** |  |  | **20** | **95.69** | **99.11** |
| **22** | ***Aralia Continentalis*** | **10** | **99.37** | **100.1** | **23** | ***Ramulus Mori*** | **10** | **95.69** | **104** | **24** | ***Citrus Unshiu Peel*** | **10** | **99.53** | **101.4** |
|  |  | **20** | **99.47** | **90.5** |  |  | **20** | **95.82** | **123.3** |  |  | **20** | **99.79** | **94.6** |
| **25** | ***Carthamus Tinctorius L.*** | **10** | **96.66** | **104.1** | **26** | ***Mori Folium*** | **10** | **102.2** | **97.7** | **27** | ***Aurantii Immatri Pericarpium*** | **10** | **97.14** | **97.8** |
|  |  | **20** | **97.03** | **115** |  |  | **20** | **92.66** | **93.9** |  |  | **20** | **102.8** | **100.9** |
| **28** | ***Rhizoma Cibotii*** | **10** | **102.4** | **112.4** | **29** | ***Houttuyniae Herba*** | **10** | **100.9** | **120.5** | **30** | ***Nelumbinis Rhizomatis Nodus*** | **10** | **93.24** | **118.7** |
|  |  | **20** | **102.7** | **105.3** |  |  | **20** | **94.22** | **106.1** |  |  | **20** | **93.27** | **98.8** |
| **31** | ***Codonopsis Pilosula*** | **10** | **93.89** | **108.7** | **32** | ***Radix Sophorae*** | **10** | **101** | **104.1** | **33** | ***Laminaria Japonica Areschoung*** | **10** | **101** | **116.9** |
|  |  | **20** | **94.17** | **113.8** |  |  | **20** | **101** | **104.6** |  |  | **20** | **91.51** | **95.8** |
| **34** | ***Cornus Officinalis*** | **10** | **97.52** | **99.3** | **35** | ***Rubi Fructus*** | **10** | **97.52** | **99.3** | **36** | ***Inonotus Obliquus*** | **10** | **97.59** | **106.4** |
|  |  | **20** | **97.58** | **95.7** |  |  | **20** | **97.58** | **95.7** |  |  | **20** | **97.59** | **107.7** |
| **37** | ***Sorbus Commixta*** | **10** | **97.63** | **94.7** | **38** | ***Schisandra chinensis*** | **10** | **103** | **136.4** | **39** | ***Hypsizigus Marmoreus*** | **10** | **101.6** | **108.6** |
|  |  | **20** | **101.5** | **97.6** |  |  | **20** | **101.5** | **142** |  |  | **20** | **101.7** | **109.2** |
| **40** | ***Herba Taraxaci*** | **10** | **102.2** | **110.4** | **41** | ***Fagopyrum Esculentum*** | **10** | **102.8** | **106.1** | **42** | ***Vitis Vinifera*** | **10** | **101.3** | **112.8** |
|  |  | **20** | **100.2** | **111.2** |  |  | **20** | **103** | **108.9** |  |  | **20** | **101.3** | **108.6** |
